# Prevalence of Infectious Pathogens Among Voluntary and Replacement Blood Donors in Pakistan: An 18-Year Experience

**DOI:** 10.7759/cureus.110515

**Published:** 2026-06-09

**Authors:** Natalia Ahmad, Hiba Asif, Faisal Sultan, Summiya Nizamuddin, Mohammad T Mahmood, Asad H Ahmad, Carly Ching, Muhammad Hamid Zaman

**Affiliations:** 1 Pathology and Laboratory Medicine, Shaukat Khanum Memorial Cancer Hospital and Research Centre, Lahore, PAK; 2 Infectious Diseases, Shaukat Khanum Memorial Cancer Hospital and Research Centre, Lahore, PAK; 3 Pathology and Laboratory Medicine, American Hospital, Dubai, ARE; 4 Pathology, Shaukat Khanum Memorial Cancer Hospital and Research Centre, Lahore, PAK; 5 Biology, Northeastern University, Boston, USA; 6 Biomedical Engineering, Boston University, Boston, USA

**Keywords:** blood donors, blood safety, hepatitis b virus, hepatitis c virus, human immunodeficiency virus, replacement donors, seroprevalence, syphilis, transfusion-transmitted infections, voluntary blood donation

## Abstract

Background: Blood transfusion is a critical therapeutic intervention; however, transfusion-transmitted infections (TTIs) continue to pose a significant threat to blood safety, particularly in low- and middle-income countries. Pakistan relies heavily on replacement blood donors, which may increase the risk of infectious disease transmission. Long-term surveillance data are essential for assessing trends in TTIs and evaluating the effectiveness of donor screening and blood safety strategies. Materials and methods: This retrospective study was conducted at the Shaukat Khanum Memorial Cancer Hospital and Research Centre in Lahore. All blood donors screened between 2006 and June 2024 were included in the analysis. Donors comprised healthy voluntary and replacement donors. Screening for hepatitis B (HBV), hepatitis C (HCV), human immunodeficiency virus (HIV), and syphilis was performed using commercially available assays according to manufacturers’ instructions. Demographic and laboratory data were retrieved from the hospital’s electronic records. Statistical analysis was carried out using chi-square tests to compare proportions and the chi-square test for trend to evaluate changes in seroprevalence over time. Results: A total of 168,401 blood donors were screened during the study period. Overall, 7,039 donors (4.17%) tested positive for at least one infectious marker. HCV demonstrated the highest seroprevalence (2.22%), followed by HBV (1.29%), syphilis (0.6%), and HIV (0.1%). The annual prevalence ranged from 1.55% to 0.82% for HBV, 3.94% to 1.34% for HCV, 0.21% to 0.06% for HIV, and 0.9% to 0.17% for syphilis. A statistically significant declining trend in positivity rates was observed for all infectious markers over the 18-year period (p < 0.05). Conclusions: This 18-year analysis demonstrates a significant reduction in TTIs among blood donors in Pakistan, reflecting improvements in donor selection and screening practices. Nevertheless, the persistently high burden of HCV underscores the need to continue strengthening voluntary blood donation programs and national blood safety initiatives.

## Introduction

Transfusion of blood and its components is a crucial therapeutic modality [1]. With 120 million units of blood donated each year globally [2], blood donors are important contributors to humanity. Although blood transfusions can save lives, there is a risk of infectious disease transmission.

Transfusion of safe blood is a universal human right, according to the World Health Organization. Safe blood means it has been thoroughly screened, is free of infections or contamination from drugs or other chemicals, is stored under proper conditions, is appropriately labeled and sealed, and is used within the specified time frame. It should be free of any blood-borne diseases, including syphilis, hepatitis B (HBV), hepatitis C (HCV), human immunodeficiency virus (HIV), or malaria [2]. HBV, HCV, and HIV can be significantly decreased with the use of a safe blood transfusion service.

In low-resource environments, where screening procedures are uneven and voluntary donor systems are inadequate, transfusion-transmitted infections (TTIs) continue to be a serious concern [2]. In low-income countries, the occurrence of TTIs is notably elevated and significantly distant from reaching a zero-risk benchmark [2].

According to the Punjab Institute of Blood Transfusion Services, over 3.5 million units of blood are collected in Pakistan annually, of which 80-85% are from replacement donors [3,4]. In contrast, voluntary donations account for only 18%.

In our study, we retrospectively investigated the seroprevalence of HBV, HCV, HIV, and syphilis among blood donors, including both voluntary and replacement donors, from Shaukat Khanum Memorial Cancer Hospital and Research Centre, a tertiary care cancer center, in Lahore, Pakistan, for a time span of 18 years (2006-2024).

## Materials and methods

Setting

This study was undertaken at Shaukat Khanum Memorial Cancer Hospital and Research Centre, with a referral base spanning Pakistan, predominantly from Punjab. The Institutional Review Board of Shaukat Khanum Memorial Hospital and Research Centre issued approval EX-22-05-25-01.

Donor population

All donors were either healthy voluntary or replacement donors (family or friends of the patient). All donors were interviewed prior to donation and excluded if they were under 18 or over 60 years of age or had hemoglobin <13.5 g/dl in males and <12.5 g/dl in females. High-risk donors were excluded via a standard questionnaire focused on obtaining a history of jaundice as well as a history of high-risk behavior for acquisition of sexual and blood-borne infections.

Testing

Over the 18-year review period, several commercial methods listed below were used to detect pathogens, and manufacturers' instructions were followed. The same methods were followed throughout the study period.

Detection of hepatitis B surface antigen (HBsAg) was carried out using a microparticle enzyme immunoassay (MEIA), AxSYM HBsAg (V2) (Abbott Diagnostics Division, Wiesbaden, Germany). Urgent testing, when required, was carried out using the IMX HBsAg (V2) (Abbott Diagnostics Division, Abbott Park, IL, USA).

Detection of anti-HCV antibodies was carried out using MEIA AxSYM HCV version 3.0 (Abbott Diagnostics Division, Wiesbaden, Germany). Urgent testing, when required, was performed using IMX HCV version 3.0 (Abbott Diagnostics Division, Abbott Park, IL, USA).

HIV-1 and HIV-2 detection was performed using IMX HIV-1/HIV-2 III Plus (Abbott Diagnostics, Wiesbaden, Germany) and AxSYM HIV-1/2 gO (Abbott Diagnostics Division, Wiesbaden, Germany).

Testing for syphilis was done via Immutrep RPR (Omega Diagnostics Limited, Alva, Scotland, UK), which is a qualitative card flocculation test utilizing cardiolipin antibody. Since the testing was done for screening purposes, a confirmatory treponemal test (e.g., fluorescent treponemal antibody absorption test or *Treponema pallidum* hemagglutination assay) was not done.

Data collection

The hospital's electronic record was accessed to gather data on the total number of specimens tested, and those reported positive for HBsAg, anti-HCV, anti-HIV-1 and -2, and RPR (rapid plasma reagin) from 2006 to June 2024. All donors screened during the study period were included in the analysis.

Statistical analysis

Two-tailed chi-square tests (also equivalent to a Z-test for proportion) were performed using GraphPad Prism version 10.0.0 for Windows (GraphPad Software, Boston, Massachusetts, USA, www.graphpad.com) to determine whether proportions of positive to negative blood tests between two groups were significant. The significance of frequency variations over time was assessed using the chi-square test for trend (Cochrane-Armitage test for trend), calculated in GraphPad Prism.

## Results

Decreasing infectious disease positivity among blood donors

**Figure 1:**
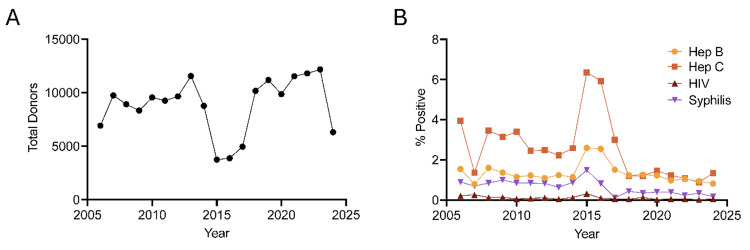
(A) Total blood donors at Shaukat Khanum Memorial Cancer Hospital and Research Centre each year from 2006 to June 2024. (B) Percent of blood donors positive for each infectious disease each year at Shaukat Khanum Memorial Cancer Hospital and Research Centre from 2006 to June 2024 Hep B: hepatitis B, Hep C: hepatitis C, HIV: human immunodeficiency virus

The frequencies (by year) of positive tests for the study period are summarized in Table 1. A total of 168401 donors were included, and repetitive donors were excluded from the analysis. The number of donors increased over the years, although there was a dip in data around 2020 (Table 1, Figure 1A). Overall, between 2006 and June 2024, 7039 (4.17%) of the total donors were positive for an infectious disease (donors with positive test results); 3740 (2.22%) were positive for HCV, 2083 (1.29%) for HBV, 170 (0.1%) for HIV, and 1046 (0.6%) for syphilis. HCV had the highest seroprevalence percentage among all the viral pathogens tested (which included HBV, HIV, and syphilis). The yearly prevalence of serologic evidence of various infectious pathogens ranged as follows: HBV 1.55-0.82%, HCV 3.94-1.34%, HIV 0.21-0.06%, and syphilis 0.9-0.17%. There was a significant downward trend in percent positivity for all viral pathogens over the years (p < 0.05, chi-square test for trends) (Figure 1B).

**Table 1:** Frequencies of positive tests for the study period (2006-June 2024) HBV: hepatitis B, HCV: hepatitis C, HIV: human immunodeficiency virus

Year	Total donors	HBV positive	%age	HCV positive	%age	HIV positive	%age	Syphilis positive	%age
2006	6926	108	1.55	273	3.94	15	0.21	69	0.9
2007	9756	78	0.79	133	1.36	26	0.26	69	0.7
2008	8925	143	1.6	309	3.46	12	0.13	76	0.85
2009	8334	114	1.37	263	3.15	12	0.14	88	1
2010	9561	111	1.16	325	3.40	6	0.06	82	0.85
2011	9256	114	1.23	227	2.45	9	0.09	79	0.85
2012	9662	107	1.10	240	2.48	11	0.11	80	0.83
2013	11562	145	1.25	258	2.23	6	0.05	74	0.64
2014	8772	101	1.15	228	2.59	12	0.13	78	0.88
2015	3740	97	2.6	238	6.36	12	0.32	59	1.5
2016	3874	99	2.55	230	5.93	4	0.10	32	0.82
2017	4953	75	1.51	149	3.0	3	0.06	6	0.12
2018	10175	125	1.23	121	1.19	5	0.05	45	0.44
2019	11187	141	1.26	136	1.21	13	0.13	39	0.35
2020	9871	121	1.23	145	1.46	3	0.03	40	0.40
2021	11542	113	0.98	143	1.23	7	0.06	46	0.39
2022	11811	124	1.05	128	1.08	8	0.065	32	0.24
2023	12175	115	0.94	109	0.89	2	0.016	41	0.34
June 2024	6319	52	0.82	85	1.34	4	0.06	11	0.17
Total	168401	2083	1.29	3740	2.22	170	0.1	1046	0.6

Differing trends between replacement and voluntary donors

**Figure 2:**
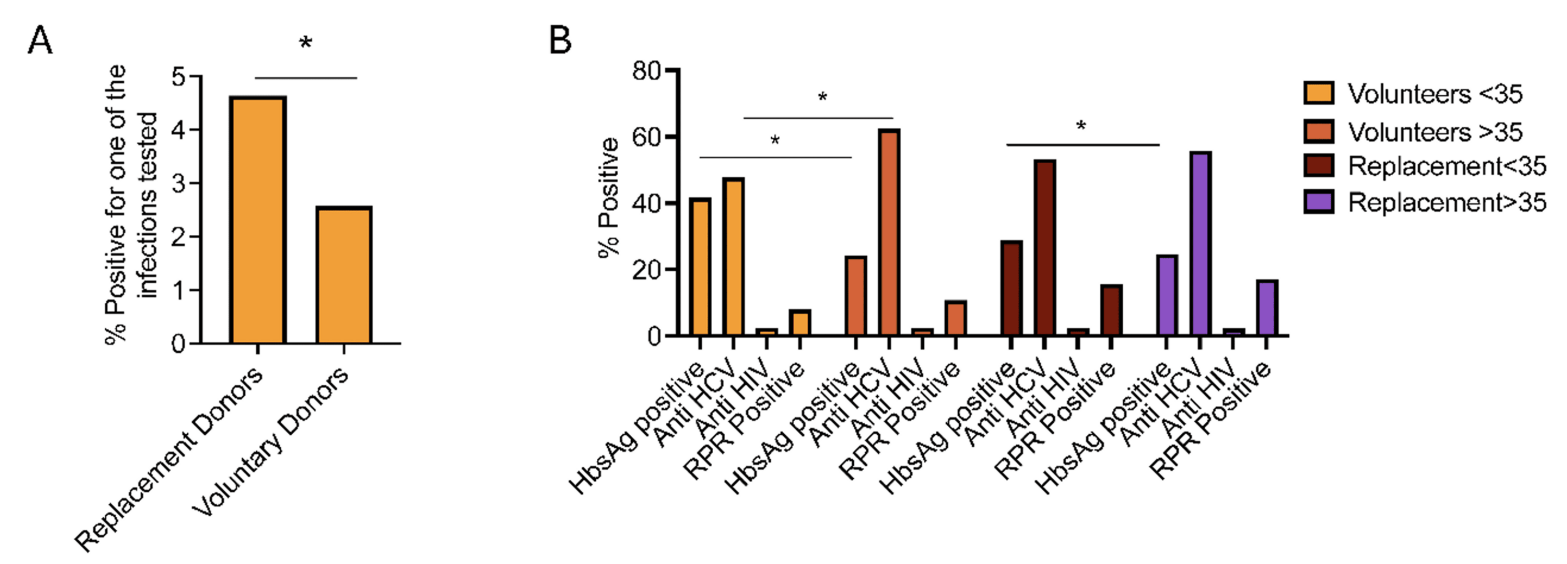
(A) Comparison of the percent positive of blood donors for one of the infectious diseases tested between replacement donors (n = 130 611) and voluntary donors (n = 37 790) across all years (2006-June 2024) at Shaukat Khanum Memorial Cancer Hospital and Research Centre. (B) Comparison of percent positive of blood donors for each infectious disease between replacement donors and voluntary donors broken down by age, across all years (2006-June 2024) Chi-square tests were performed to compare the significance between the two groups. * represents p < 0.05. HBsAg: hepatitis B surface antigen, HCV: hepatitis C, HIV: human immunodeficiency virus

Notably, the overall frequency of blood tests positive for infectious diseases was significantly higher among replacement donors than among voluntary donors (4.64% (6066/130,611) vs. 2.57% (973/37,790), respectively; χ² = 312.9, p < 0.001) (Figure 2A). Across age groups, donors aged ≥35 years had a significantly higher prevalence of infectious diseases than those aged <35 years (5.50% (1147/20,841) vs. 3.99% (5892/147,560), respectively; χ² = 103.7, p < 0.001).

Among all positive screening results in voluntary donors, younger individuals (<35 years) had a significantly lower HCV seroprevalence than those aged ≥35 years (47.8% vs. 62.4%, respectively; χ² = 10.7, p = 0.001) (Table 2). In contrast, HBV was significantly more prevalent among younger voluntary donors than among those aged ≥35 years (41.6% vs. 24.2%, respectively; χ² = 16.2, p < 0.001) (Figure 2B). The seroprevalence of syphilis and HIV did not differ significantly between age groups among voluntary donors.

**Table 2:** Trends of positive donors (replacement and voluntary donors) HBV: hepatitis B, HCV: hepatitis C, HIV: human immunodeficiency virus, RPR: rapid plasma reagin

Group	Total screened positive	HBV positive		HCV positive		HIV positive		RPR positive	
	Number	Number	%age	Number	%age	Number	%age	Number	%age
Voluntary donors <35 years	816	340	41.6	390	47.8	20	2.5	66	8.0
Voluntary donors >35 years	157	38	24.2	98	62.4	4	2.5	17	10.8
Replacement donors <35 years	5076	1461	28.8	2699	53.2	123	2.4	793	15.6
Replacement donors >35 years	990	244	24.6	553	55.8	23	2.3	170	17.2

Among replacement donors, HBV prevalence was significantly higher in younger donors (<35 years) than in donors aged ≥35 years (28.8% vs. 24.6%, respectively; χ² = 6.8, p = 0.009) (Figure 2B). However, the prevalences of HCV, syphilis, and HIV did not differ significantly between age groups among replacement donors.

## Discussion

Transfusion of blood and blood components is a crucial lifesaving procedure that aids numerous patients worldwide. Simultaneously, the transfusion of infected blood can spread blood-borne infectious agents. The risk of TTI per transfused blood unit is estimated at approximately 1% [5]. This estimation represents a considerable risk for blood-borne diseases, as several of these infections are severe, life-threatening, and either incurable or challenging to treat. In the past 20 years, the implementation of HBV vaccination has significantly reduced the incidence of TTIs across many countries. Education is a crucial element that can significantly reduce the incidence of infectious pathogens in blood donors, primarily by lessening risky behaviors [6].

Despite recent epidemiological reports indicating notable advances in ensuring blood safety through comprehensive system reforms [7] and the adoption of hemovigilance practices [8], the risk of TTI remains a concern in Pakistan. In our current study at Shaukat Khanum Memorial Cancer Hospital and Research Centre, 168,401 blood donors were screened for infectious pathogens over a period of 18 years (2006-2024). The overall prevalence of infectious pathogens in donated blood was 4.17%, with 2.22%, 1.29%, 0.1%, and 0.6% for HCV, HBV, HIV, and syphilis, respectively. We have summarized the prevalence of infectious pathogens in Pakistan from local studies in Table 3 and compared the results with our study. Few local studies showed comparable results with our study, with pathogen prevalence rates of 4% [9],4.61% [10], and 3.24% [11], while some of the local studies showed higher percentages of infectious pathogens: 6.5% [3], 9.06% [12], and 5.46% [13].

**Table 3:** Frequency of infectious pathogens in blood donors in published studies from Pakistan HBsAg: hepatitis B surface antigen, HCV: hepatitis C, HIV: human immunodeficiency virus, RPR: rapid plasma reagin, VDRL: venereal disease research laboratory

Reference	Years	Location	Total number of donors	%age of infectious pathogens	HBsAg % age positive	Anti-HCV % age positive	Anti-HIV % age positive	VDRL/RPR %age positive
Ahmad et al. (2019) [9]	2012-2016	Lahore, Punjab	79,774	4	0.9	1.7	0	1.1
Saba et al. (2021) [10]	2016-2020	Peshawar, Khyber Pakhtunkhwa	41,817	4.61	1.95	1.38	0.23	0.91
Rauf et al. (2019) [11]	2018-2019	Faisalabad, Punjab	6,594	3.24	0.18	1.12	0.18	1.1
Ehsan et al. (2020) [3]	2010-2020	Punjab, Sindh and KPK	1,606,645	6.5	2.04	2.44	0.038	1.1
Jamal et al. (2023) [12]	2017-2021	Punjab and Sindh	477,938	9.06	3.06 3.94	5.89 5.85	0.37 0.29	2.12 4.16
Saeed et al. (2017) [13]	2014-2015	Lahore, Punjab	18,274	5.46	1.1	2.62	0.02	1.55
Present study	2006-2024	Punjab and KPK	168,401	4.17	1.29	2.34	0.1	0.6

The prevalence of infectious pathogens among blood donors in published studies from the surrounding regions is shown in Table 4. Notably, the % positive for HBV (Avg. 1.7% to 1.29%), HIV (Avg. 0.16% to 0.1%), and syphilis (0.3% to 0.6%) are similar to those in our study. Still, the seroprevalence of HCV is typically much lower in the local region (Southeast Asia) than in our study in Pakistan (avg. 0.8% vs. 2.22%). Notably, the average for HCV among published studies in Pakistan is also higher, at 2.9%.

**Table 4:** Frequency of infectious pathogens in blood donors in published studies from the region HBsAg: hepatitis B surface antigen, HCV: hepatitis C, HIV: human immunodeficiency virus, RPR: rapid plasma reagin, VDRL: venereal disease research laboratory

Reference	Years	Location	Number	HBsAg % positive	Anti-HCV % positive	Anti-HIV % positive	VDRL/RPR% positive
Thakur et al. (2025) [14]	2025	Delhi, India	42158	1.04	0.42	0.22	0.37
Varma et al. (2019) [15]	2015-2018	Indore, India	45,704	1.29	0.07	0.076	NR
Fatima et al. (2016) [16]	2010-2016	Nizamabad District, India	55291	0.69	0.01	0.20	0.03
Gudum et al. (2024) [17]	2010-2019	Malaysia	7,329	1.14	2.69	0.42	0.39
Shrivastava et al. (2023) [18]	2001-2016	India	57942	1.8	0.42	0.2	0.31
Mesbahzadeh et al. (2021) [19]	2009-2019	Iran	682,171	0.25	0.027	0.005	0.036
Minshawi et al. (2024) [20]	2017-2022	Saudi Arabia	40,287	6.1	0.4	0.06	0.34
Present study	2006-2024	Punjab and KPK	168401	1.29	2.34	0.1	0.6

Another significant finding of the present study was that the prevalence of infectious pathogens in replacement donors was higher (4.64%) than in voluntary blood donors (2.57%; Figure 2A). Many other international and local studies showed similar findings [9-16,18]. The higher prevalence of infectious pathogens among replacement blood donors compared with voluntary donors is likely due to differences in motivations and screening practices between the groups. Replacement donors, often donating to help a specific patient, may be donating under pressure and less likely to have previously donated blood. In contrast, voluntary donors are generally motivated by altruism and are more likely to be part of a regular donor pool with established screening protocols [13,14].

Our study found a downward trend in HBV, HCV, HIV, and syphilis positivity (Figure 1B). The decreasing trend in the prevalence of TTI pathogens among blood donors is primarily due to improved donor screening [21], increased public awareness, and the implementation of more sensitive and specific testing methods [22,23]. These factors have collectively reduced the risk of transmitting infections like HIV, HBV, HCV, and syphilis through blood transfusions. For example, our blood bank has implemented more stringent donor selection criteria, including detailed health questionnaires and physical examinations to identify individuals at higher risk of carrying TTIs. Public awareness campaigns have increased knowledge about infectious pathogens transmitted through blood transfusion, their modes of transmission, and the importance of safe blood practices. Public awareness campaigns also encourage voluntary blood donation, which is associated with a lower prevalence of TTIs.

This study had a few limitations. First, it was conducted at a single tertiary care cancer center, which may limit the generalizability of the findings to Pakistan's overall population. Second, detailed information on donor risk factors, vaccination status, socioeconomic background, and behavioral practices was unavailable, limiting further analysis of factors associated with TTIs.

## Conclusions

A significant reduction in TTIs among blood donors was observed over the 18-year study period, reflecting improvements in donor selection, screening practices, public awareness, and overall blood safety measures in Pakistan. The declining trends in HBV, HCV, HIV, and syphilis positivity rates demonstrate the positive impact of strengthened transfusion services and implementation of more rigorous screening protocols over time.

Despite these encouraging findings, HCV remained the most prevalent infectious marker throughout the study period, highlighting the persistent burden of HCV in the donor population. These findings emphasize the need for continued strengthening of voluntary non-remunerated blood donation programs, enhanced donor education and pre-donation screening, and consistent implementation of high-quality screening strategies and surveillance systems to further improve blood safety in Pakistan.
